# Nutrition as a missing piece in the development of youth male soccer players: a scoping review and future directions

**DOI:** 10.5114/biolsport.2025.151654

**Published:** 2025-09-09

**Authors:** Diogo V. Martinho, Oliver Gonzalo-Skok, Karim Chamari, Adam Field, Filipe Manuel Clemente, André Rebelo, Élvio R. Gouveia, Vitor Hugo Teixeira, Piotr Zmijewski, Pedro Mendes, Pedro Campos, Hugo Sarmento

**Affiliations:** 1 University of Coimbra, Faculty of Sport Sciences and Physical Education, Coimbra, Portugal; 2 Interactive Technologies Institute, Laboratory of Robotics and Engineering Systems, Funchal, Portugal; 3 Department of Communication and Education, Universidad Loyola Andalucía, Seville, Spain; 4 Naufar, Wellness and Recovery Center, Qatar; 5 Department of Sport and Exercise Science, Institute of Sport, Manchester Metropolitan University, Manchester, UK; 6 Escola Superior Desporto e Lazer, Instituto Politécnico de Viana do Castelo, Rua Escola Industrial e Comercial de Nun’Álvares, Viana do Castelo, Portugal; 7 Sport Physical Activity and Health Research & Innovation Center, Viana do Castelo, Portugal; 8 Gdansk University of Physical Education and Sport, Gdańsk, Poland; 9 Universidade Lusófona, CIDEFES, Centro de Investigação em Desporto, Educação Física e Exercício e Saúde, Lisboa, Portugal; 10 COD, Center of Sports Optimization, Sporting Clube de Portugal, Lisboa, Portugal; 11 Department of Physical Education and Sport, University of Madeira, Funchal, Portugal; 12 Faculty of Nutrition and Food Sciences, University of Porto (FCNAUP), Porto, Portugal; 13 Research Centre in Physical Activity, Health and Leisure (CIAFEL), Faculty of Sports, University of Porto (FADEUP), Porto, Portugal; 14 Laboratory for Integrative and Translational Research in Population Health, ITR, Porto, Portugal; 15 Institute of Sport – National Research Institute, 01-982 Warsaw, Poland; 16 Polytechnic of Coimbra, Coimbra Health School, Dietetics and Nutrition, Coimbra, Portugal; 17 Department of Informatics Engineering and Interactive Media Design, University of Madeira, Funchal, Portugal; 18 WoWSystems Informática Lda, Funchal, Portugal; 19 University of Coimbra, CIPER, FCDEF, Coimbra, Portugal

**Keywords:** Carbohydrates, Energy, Dietary intake, Talent development, Soccer demands

## Abstract

The literature on nutrition in soccer has extensively focused on adult male and female soccer players, while knowledge regarding nutritional issues in youth soccer players remains limited. This review aims to summarize the findings related to nutritional habits and ergogenic aid practices among male youth soccer players. Following the Preferred Reporting Items for Systematic Reviews and Meta-Analyses Extension for Scoping Reviews (PRISMA-ScR) Checklist, four databases were consulted on September 17, 2024. Studies that included youth soccer players and examined daily energy intake or expenditure, as well as the effects of ergogenic aids on performance, met the eligibility criteria for this review. In total, 42 studies were considered. Among these, 22 studies focused on energy expenditure and dietary intake, while 20 studies investigated the effects of ergogenic aids on performance. Overall, a negative energy balance was observed; however, after adjusting for the underestimation of energy intake, an adequate intake compared to energy expenditure was found in this review. Additionally, carbohydrate intake tended to decrease with age, while protein intake remained stable throughout adolescence. The topic of macronutrient periodization in youth players requires further research, as no data is currently available regarding macronutrient intake. Additionally, data concerning the effects of ergogenic aids on performance is limited due to variability in methodological procedures. Nevertheless, caffeine and creatine appear to have a positive effect on physical capabilities. This review provides reference data for nutritionists working with youth soccer players and highlights the need for future research in this area.

## INTRODUCTION

Youth soccer clubs and academies strive to develop well-rounded players, focusing on technical, tactical, physical, physiological, and social skills [1, 2]. As a result, more players are likely to progress to the senior level, which is linked to both sporting success and financial gains for the clubs [1, 3]. Soccer academies typically structure their programmes by chronological age, highlighting the importance of various stakeholders—such as coaches, scouts, medical staff, and family members—in supporting the development of youth soccer players [4, 5]. Nutrition is a crucial aspect of youth soccer development, yet it is often overlooked or undervalued in comparison to sports science and medical disciplines within soccer academies [6].

Adequate nutrition enables youth soccer players to meet training demands similar to those of adult elite players [7–9]. Adolescence is a period of significant changes in body size, composition, and biological maturation [10, 11]. As a result, adolescents have increased nutritional requirements, not only to support their training but also to meet the energy demands of growth and maturation [7, 12]. Therefore, nutritional guidelines designed to optimize performance in adult soccer [13] should not be directly applied to youth players. Summarizing the nutritional evidence to support the development of youth soccer players is a crucial task for clubs and academies.

A previous review covered a broad range of topics, including nutritional intake and energy balance, ergogenic aids and supplements, hydration, the influence of Ramadan, Vitamin D status, female players, and nutrition knowledge. It also highlighted several nutritional concerns within youth soccer academies, including negative energy balance, inadequate carbohydrate intake, and poor adherence to periodized nutrition strategies [14]. However, drawing robust conclusions from these findings is challenging due to the limited search strategy. For instance, a two-year gap existed between the implementation of the search strategy and publication of the review article, during which new evidence may have emerged, rendering some of the findings outdated. This time lag can also affect the practical application of the review’s findings, especially in fast-moving areas like youth soccer and nutrition. Moreover, youth soccer players are currently receiving advice from non-specialists within soccer academies, such as coaches, physiotherapists, sports science staff, and catering personnel, who may lack nutritional expertise. This can negatively impact growth, maturation, and overall performance [6, 15]. Updating nutritional practices for youth players is a critical aspect of talent development. A scoping review focused on dietary practices and the effects of ergogenic aids on performance could offer valuable insights and future directions for both practitioners and researchers.

Recognising the critical role of nutrition in supporting growth, maturation, and soccer performance in youth, this scoping review aims to: (1) describe nutritional and energy intake; (2) evaluate the acute and chronic effects of ergogenic supplements on performance; and (3) identify gaps in the literature to provide suggestions for future research.

## MATERIALS AND METHODS

This review adhered to established methodological frameworks, including the Cochrane guidelines [16], the PRISMA 2020 guidelines [17] and corresponding extensions for scoping reviews [18]. The protocol was registered by the first author (DVM) on the Open Science Framework (https://doi.org/10.17605/OSF.IO/VN2T6).

### Eligibility criteria

Original manuscripts or ahead-of-print articles written in English, Portuguese, or Spanish were considered for the review without date restrictions. Inclusion criteria were defined using the Participants, Intervention, Comparator, Outcomes, and Study Design (PICOS) framework:

1. **Participants:** Male youth players (under-9 to under-23) classified as Tier 3 (highly trained/national level) or Tier 4 (elite/international level) according to the participation classification frame-work [19].
2. **Intervention:** Studies describing dietary or nutritional practices or examining the effects of ergogenic aids on performance.
3. **Comparator:** Not applicable for observational studies; placebo or control groups in interventional designs.
4. **Outcomes:** Measures of energy and macronutrient intake, as well as energy expenditure (resting, exercise, total daily).
5. **Study Design:** Observational and interventional studies.

Studies that included multiple sports or involved recreational, university, or female players were excluded from this review. An extensive review focusing on female soccer players (both adult and youth) has been published elsewhere [20].

### Information sources and search strategy

On September 17, 2024, we conducted a comprehensive search across four electronic databases: PubMed, Scopus, SPORTDiscus, and Web of Science. The search strategy employed was as follows: ((nutrition* OR energ* OR intake OR expenditure OR diet* OR carbohydrate OR glucose OR protein OR collagen OR fat OR ketone* OR antioxidant* OR “vitamin D” OR polyphenol* OR fruit OR creatine OR caffeine OR nitrate* OR beetroot OR “beta alanine” OR “sodium bicarbonate”) AND (soccer OR soccer) AND (academy OR youth OR adolescent* OR young* OR “young athlete”)). Additionally, we reviewed the references from a previous systematic review [14] to identify any additional relevant manuscripts for inclusion.

### Selection process

EndNote™ 21.0 (Clarivate™, Philadelphia, Pennsylvania, USA) was used to facilitate the identification, screening, and inclusion steps of our systematic review. Duplicate records were initially removed automatically by EndNote’s “Find Duplicates” feature. Subsequently, the first and last authors (DVM/HS) manually confirmed and resolved any remaining duplicates. An initial screening was performed based on titles and abstracts, followed by full-text assessments to ensure studies met the inclusion criteria. Two researchers (DVM/AR) conducted the screenings, and in cases of disagreement, a third author (HS) was consulted to reach a consensus.

### Data extraction and items

Microsoft Excel^®^ (version 2501, Microsoft) was used to systematically collect pertinent information from each manuscript. This process was conducted by two authors (DVM/AR), with a third researcher cross-validating the data. We developed two comprehensive datasets aligned with the study’s objectives: one focusing on dietary practices and energy intake, and the other on the effects of ergogenic aids. Data on dietary intake and energy expenditure, both absolute and relative, were collected. Information regarding ergogenic aids— including study type, supplement, dosage, timing, formulation, and performance outcomes—was also gathered. Additionally, details such as country, sample size, competitive level, methodologies, and main results were compiled for both groups of studies. When relevant information was not available in the manuscripts, we contacted the corresponding authors for clarification. Studies lacking essential inclusion criteria information, such as age or competitive status, were excluded. To extract graphical data, we employed specialized software, GetData Graph Digitizer, which is effective for collecting means and standard deviations from graphs (getdata-graph-digitizer.software. informer.com). This tool facilitated the accurate digitization of data from visual representations in the included studies [21].

### Data analysis

The frequency of published studies, categorized by the country of competition and the age of youth participants, is presented in bar charts for research focusing on energy/dietary outputs and ergogenic aids.

To assess overall energy intake, carbohydrate and protein consumption, energy expenditure, and resting energy expenditure, the meta-analysis considered the mean, standard deviation, and sample size. An integrative approach was employed to manage multiple means and standard deviations from the same study, given the crosssectional design of the studies included in this review [22].

Overall differences between energy intake and energy expenditure were calculated using two approaches: 1) the reported values of energy intake, and 2) adjusted values of energy intake increased by 15% [23]. Mean differences, standard errors of mean differences, and sample sizes were included in the meta-analysis. Since standard deviations of within-participant differences between energy intake and energy expenditure were not reported, they were estimated based on the correlation coefficients extracted from a previous review [24]. A reductionist approach was employed to consolidate multiple differences between energy intake and energy expenditure within the same study [22]. In all meta-analyses conducted, publication bias was graphically inspected using a funnel plot and statistically verified with Egger’s test. The trim-and-fill method proposed by Duval and Tweedie was utilized to adjust for potential publication biases [25]. A random-effects model was applied to accommodate variability across different methods used to assess the variables of interest [26]. The relationship between age and the variables was interpreted using the following classifications [27]: trivial (< 0.100), small (0.100 to 0.299), moderate (0.300 to 0.499), large (0.500 to 0.699), very large (0.700 to 0.899), and nearly perfect (0.900 to 0.999). The comparison of mean differences in energy intake, considering match days, training sessions, or rest days, was interpreted according to the following classifications [27]: d < 0.20 (trivial), 0.20 < d < 0.59 (small), 0.60 < d < 1.19 (moderate), 1.20 < d < 1.99 (large), 2.00 < d < 3.99 (very large), and d > 4.00 (nearly perfect).

The effects of ergogenic aids on performance were expressed as a percentage using the formula [((mean caffeine – mean placebo) / ((mean caffeine + mean placebo) ÷ 2) × 100].

All statistical analyses were conducted using Comprehensive Meta-Analysis software (version 2; Biostat, Englewood, NJ, USA) and GraphPad Prism (version 5.00 for Windows, GraphPad Software, San Diego, California, USA, www.graphpad.com), with statistical significance set at *p* < 0.05.

### Risk of bias

Two independent experiment authors (DVM/AR) analyzed the quality of individual studies. A third author was consulted to solve the disagreements (HS). The risk of bias was assessed using two different tools according to the study design: (1) the Quality Assessment Tool for Observational Cohort and Cross-Sectional Studies [28]; (2) The PEDro scale is an 11-item validated tool to measure the risk of bias and statistical reporting of clinical trials (https://pedro.org.au/english/resources/pedro-scale/).

The first tool included fourteen items about the research question, study population, groups recruited from the same population and uniform eligibility criteria, sample size justification, exposure assessed before measurement outcome, sufficient timeframe to observe an effect, different levels of the exposure effect, exposures measurement, repeated exposure assessment, outcomes measurement, blinding of outcomes assessors, follow-up rate and statistical analysis. For this review, nine items from the assessment tool were deemed relevant for evaluating the quality of each study. Each item was interpreted individually.

The 11-item PEDro scale presents questions about the eligibility criteria, group allocation, group similarities at baseline, blinding procedures, completion rates of the outcome measures, statistical analyses, and reporting of outcome measures. Each item was assigned a value of ‘yes’ (corresponding to 1 point) or ‘no’ (corresponding to 0 points). The first item is not considered to calculate the PEDro score. The quality of the interventional studies was interpreted using the following criteria [29]: 0–3 points was considered “poor” quality, 4–5 points was considered “fair” quality, 6–8 points was considered “good” quality, and 9–10 points was considered “excellent” quality. No studies were excluded based on their assessed risk of bias.

## RESULTS

### Study identification and selection

The systematic review process identified 7,744 records across four electronic databases. After removing 3,364 duplicates, 4,380 records were screened based on titles and abstracts. This screening led to the exclusion of 4,257 records, leaving 123 studies for full-text assessment. Eighty-four studies were excluded for various reasons, including: competitive level not meeting the highly trained/national or elite/international standards (*n* = 23), inclusion of adult players (*n* = 21), lack of reported nutritional intake or examination of ergogenic aids’ effects on performance (*n* = 10), missing information on age or competitive level, even after contacting authors (*n* = 18), focus solely on diet or meal effects (*n* = 5), studies involving American, Australian, or futsal players (*n* = 5), combination of different sports in one study, presentation of duplicate data in one study.

Consequently, 39 studies were initially included in the review. Further examination of reference lists from a previous review [14] and the current review’s included papers led to the addition of three more eligible studies. Thus, a total of 42 studies were incorporated into this review, as depicted in the PRISMA flow diagram.

### Study characteristics

Table 1 presents the study and sampling characteristics, including country, sample size, age, height, weight, and topic of analysis. Among the reviewed studies, twenty-two focused on the energy expenditure and dietary intake of youth soccer players, while twenty others examined the effects of ergogenic aids on performance.

**TABLE 1:** Characteristics of the participants examined in each study and the main topic investigated.

Study	Country	Sample size (N)	Age (yrs)	Height (m, cm)	Weight (kg)	Topic
Zeederberg et al. [51]	South Africa	20	Under-19	NR	NR	Ergogenic aid

Sanz et al. [30]	Puerto Rico	8	17 ± 2	170 ± 7	63 ± 3	Energy expenditure and dietary intake

Mujika et al. [52]	Spain	17	20 ± 1	180 ± 6	75 ± 6	Ergogenic aid

LeBlanc et al. [46]	France	180	13–16	NR	58 ± 8	Energy expenditure and dietary intake

Ostojic et al. [53]	Servia	20	17 ± 2	175 ± 9	64 ± 6	Ergogenic aid

Iglesias-Gutiérrez et al. [40]	Spain	33	14–16	1.76	65	Energy expenditure and dietary intake

Murph and Jeanes [41]	England	35	16–19	1.75 ± 0.07	72 ± 4	Energy expenditure and dietary intake

Caccialanza et al. [42]	Italy	43	16 ± 1	1.75 ± 0.05	70 ± 7	Energy expenditure and dietary intake

Iglesias-Gutiérrez et al. [43]	Spain	22	14–16	1.78	63	Energy expenditure and dietary intake

Arent et al. [54]	US	24	20 ± 2	175.5 ± 7.3	74.8 ± 7.3	Ergogenic aid

Pereira et al. [55]	Brazil	15	15 ± 2	1.69 ± 0.07	58.8 ± 9.2	Ergogenic aid

Holway et al. [47]	Argentina	91	14 yrs: 15 ± 0.2 15 yrs: 15 ± 0.1 16 yrs: 17 ± 0.2 17 yrs: 18 ± 0.2 18 yrs: 19 ± 0.2 19 yrs: 20 ± 0.2	14 yrs: 171 ± 7 15 yrs: 173 ± 7 16 yrs: 173 ± 6 17 yrs: 174 ± 7 18 yrs: 178 ± 9 19 yrs: 175 ± 7	14 yrs: 65 ± 9 15 yrs: 69 ± 10 16 yrs: 68 ± 8 17 yrs: 69 ± 9 18 yrs: 75 ± 8 19 yrs: 72 ± 5	Energy expenditure and dietary intake

Russel and Pennock [32]	England	10	17 ± 1	1.72 ± 0.01	68 ± 2	Energy expenditure and dietary intake

Iglesias-Gutiérrez et al. [48]	Spain	87	18 ± 2	179 ± 6	73 ± 7	Energy expenditure and dietary intake

Russel et al. [56]	England	15	18 ± 1	1.77 ± 0.05	70 ± 2	Ergogenic aid

Bortolotti et al. [66]	Brazil	9	15 ± 2	1.72 ± 0.05	61 ± 5	Ergogenic aid

Jordan et al. [57]	U.S.	14	14 ± 1	172 ± 5	61 ± 5	Ergogenic aid

Petterson et al. [58]	Norway	22	18 ± 1	NR	72 ± 7	Ergogenic aid

Russel et al. [59]	England	10	16 ± 1	1.74 ± 0.02	65 ± 2	Ergogenic aid

Briggs et al. [33]	England	10	15 ± 0.3	1.70 ± 0.06	58 ± 8	Energy expenditure and dietary intake

Elizondo et al. [49]	Mexico	72	15–20	173 ± 1	65 ± 2	Energy expenditure and dietary intake

Harper et al. [67]	England	8	16 ± 1	1.73 ± 0.05	69 ± 5	Ergogenic aid

Jastrzębska et al. [69]	Poland	36	18 ± 1	NR	71 ± 7	Ergogenic aid

Bettonviel et al. [40]	Dutch	15	17 ± 1	178.2 ± 6.2	69 ± 6	Energy expenditure and dietary intake

Naughton et al. [9]	England	59	U13/U14: 13 ± 0.6 U15/U16: 14 ± 0.5 U18: 16 ± 0.5	U13/U14: 157 ± 11 U15/U16: 173 ± 8 U18: 180 ± 7	U13/U14: 45 ± 7 U15/U16: 60 ± 8 U18: 71 ± 8	Energy expenditure and dietary intake

Yãnez-Silva et al. [61]	Brazil	19	17 ± 1	177 ± 5	70 ± 3	Ergogenic aid

Granja et al. [34]	Portugal	10	15 ± 0.4	1.77 ± 0.05	70 ± 3	Energy expenditure and dietary intake

Hosseinzadeh et al. [45]	Iran	40	15 ± 1	175 ± 7	61 ± 7	Energy expenditure and dietary intake

Raizel et al. [35]	Brazil	19	21 ± 2	1.75 ± 0.09	72 ± 8	Energy expenditure and dietary intake

Azevedo et al. [69]	Brazil	8	16 ± 1	1.78 ± 0.06	71 ± 4	Ergogenic aid

Ellis et al. [62]	England	15	16 ± 1	177 ± 5	70 ± 8	Ergogenic aid

Ersoy et al. [36]	Turkey	26	16 ± 1	175 ± 7	67 ± 6	Energy expenditure and dietary intake

Rodriguez-Giustiniani et al. [63]	England	18	18 ± 2	178 ± 5	73 ± 6	Ergogenic aid

Skalska et al. [71]	Poland	36	18 ± 1	NR	71 ± 7	Ergogenic aid

Noronha et al. [51]	Brazil	73	17 ± 1	1.82 ± 0.10	66 ± 7	Energy expenditure and dietary intake

Hannon et al. [37]	England	24	U12/U13: 12 ± 0.4 U15: 15 ± 0.2 U18: 18 ± 0.4	U12/U13: 157 ± 4 U15: 174 ± 6 U18: 181 ± 5	U12/U13: 43 ± 5 U15: 57 ± 6 U18: 73 ± 8	Energy expenditure and dietary intake

Nobari et al. [64]	Iran	29	15 ± 0.3	Not reported	Not reported	Ergogenic aid

Carter et al. [39]	England	24	18 ± 2	1.80 ± 0.07	77 ± 8	Energy expenditure and dietary intake

Martinho et al. [38]	Portugal	25	15 ± 0.3	171 ± 7	62 ± 7	Energy expenditure and dietary intake

Stables et al. [31]	England	8	13 ± 0.2	162 ± 7	51 ± 8	Energy expenditure and dietary intake

Stables et al. [46]	England	48	U12: 12 ± 0.1 U13: 13 ± 0.2 U14: 14 ± 0.1 U15/U16: 16 ± 0.3 U18: 17 ± 0.3 U23: 19 ± 2	U12: 154 ± 4 U13: 162 ± 9 U14: 169 ± 9 U15/U16: 184 ± 5 U18: 185 ± 5 U23: 186 ± 7	U12: 45 ± 7 U13: 49 ± 8 U14: 58 ± 10 U15/U16: 70 ± 7 U18: 70 ± 7 U23: 77 ± 7	Energy expenditure and dietary intake

Jafari et al. [65]	Iran	12	16–17	174.2 ± 8.4	63 ± 8	Ergogenic aid

Kuru et al. [66]	Turkey	20	20 ± 2	176.3 ± 5.9	74 ± 6	Ergogenic aid

NR (Not Reported); U (Under); yrs (years).

### Energy expenditure and dietary intake

For studies on energy expenditure and dietary intake, eight were conducted in England (approximately 37%), and three were developed in Spain (around 14%), as illustrated in Figure 2 (panel A). The sample sizes varied from 8 to 180 players, with a total pooled sample size of 947 players. Specifically, two studies included fewer than 10 players [30, 31], while nine studies examined between 10 and 30 players [32–40]. Six studies had sample sizes ranging from 30 to 50 players [41–46], while five studies utilized larger samples (> 70 players) [47–51]. Additionally, one study included 59 players [9]. A considerable number of studies included mid-adolescent (15 studies) and late adolescent (19 studies) players (Figure 2, panel B).

**FIG. 1:**
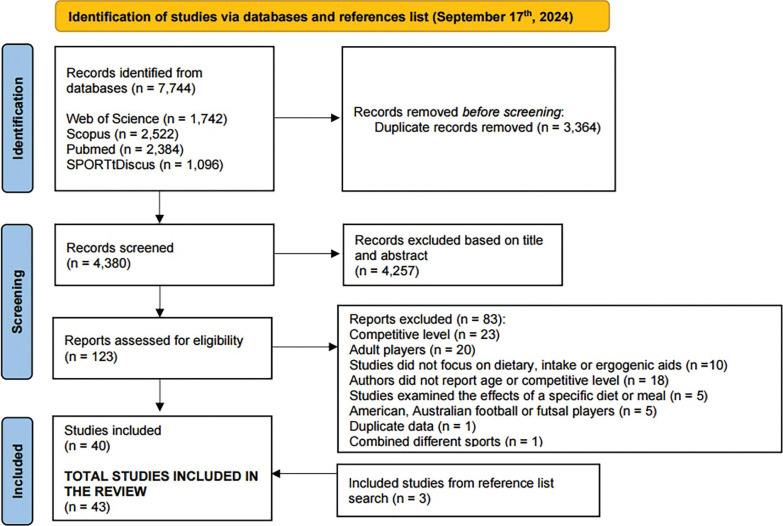
Prisma flow diagram of the systematic procedure for studies selection.

**FIG. 2:**
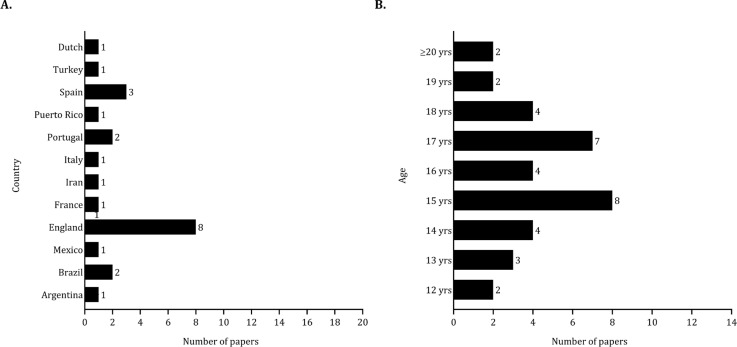
Number of studies published by country (panel A) and age (panel B) considering the dietary intake and energy expenditure.

### Ergogenic aids

**FIG. 3:**
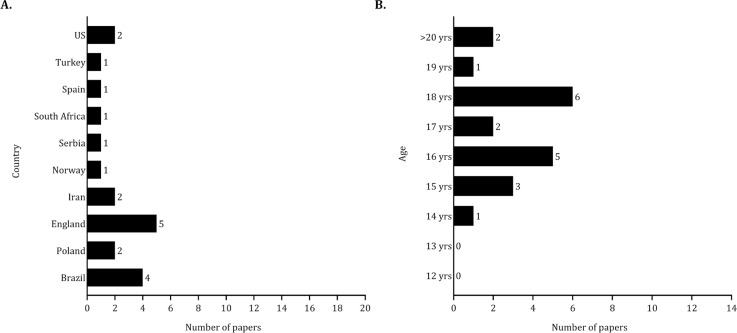
Number of studies published by country (panel A) and age (panel B) considering the effects of ergogenic aids on performance.

The research on ergogenic aids was primarily conducted in England (about 24%) and Brazil (approximately 19%), as shown in Figure 3 (panel A). The sample sizes in studies examining the effects of ergogenic aids on performance ranged from 8 to 36 players, with a total pooled sample size of 389 players. Fifteen studies involved between 10 and 30 players [52–66]. Additionally, three studies included fewer than 10 players [67–69], while two studies involved 36 players [70, 71]. Most of the samples included in studies examining the effects of ergogenic aids on performance primarily involved late adolescent players (16 studies) as shown in Figure 3 (panel B).

### Results of individual studies and statistical analysis

#### Energy expenditure and dietary intake

Table 2 summarizes the methodological approaches used in dietary intake and energy assessments. Fourteen studies focused on youth soccer players during the in-season, while three studies concentrated on the pre-season. Additionally, four studies did not specify the assessment period, and none focused on the off-season. Twelve studies (~ 55%) examined dietary intake using food diaries. The food was weighed and documented in three studies (~14%), while 24-hour recalls were utilized in two studies (~9%). Five studies employed a combination of different methodologies (~23%). Resting energy expenditure was predicted using various equations in seven studies (~33%), while indirect calorimetry was employed in two studies. Eleven studies estimated the daily energy expenditure. Daily records or factorial method (i.e., multiplying the resting energy expenditure by a specific factor for physical activity levels) were used in seven studies. The estimation of energy expenditure with the doubly labelled water method was reported in two studies.

**TABLE 2:** Methodological approaches used in studies examining energy intake and energy expenditure.

Study	Period of season	Nutritional and energetic assessment			Analysis	

		Dietary instrument	Resting energy expenditure	Daily energy expenditure	By playing position	According to competitive context
Sanz et al. [30]	NR	12-day food diary		Daily record		
LeBlanc et al. [47]	Pre-season	5-day food diary				
Iglesias-Gutiérrez et al. [49]	In-season	6-day weighted food diary	Schofield equation			
Murph and Jeanes [42]	In-season	7-day food diary				
Caccialanza et al. [43]	NR	4-day food diary		Factorial method		
Iglesias-Gutiérrez et al. [44]	In-season	6-day weighted food diary				
Holway et al. [48]	NR	24 h recall (5 days)	NR	NR		
Russel and Pennock [32]	In-season	7-day food diary	Harris-Benedict equation	Factorial method		×
Iglesias-Gutiérrez et al. [44]	In-season	6-day food diary			×	
Briggs et al. [33]	In-season	7-day weighted food diary, 24 h recall	Schofield equation	Accelerometery		×
Elizondo et al. [50]	In-season	4-day weighted food diary	FAO/WHO/UNU equation	Factorial method		
Bettonviel et al. [40]	NR	24 h recall (4 days)				×
Naughton et al. [9]	Pre-season	7-day food diary				
Granja et al. [34]	In-season	9-day food diary				×
Hosseinzadeh et al. [45]	NR	24 h recall (3 days)				
Raizel et al. [35]	Pre-season	3-day food diary			×	
Ersoy et al. [36]	Pre-season	3-day food diary				
Noronha et al. [51]	In-season	3-day food diary	Harris-Benedict equation	Factorial method		
Hannon et al. [37]	In-season	7-day food photographic diary, 24 h recall	Indirect calorimetry	Doubly labelled water		
Carter et al. [39]	In-season	7-day food photographic diary, 24 h recall	Indirect calorimetry			×
Martinho et al. [38]	In-season	3-day food diary	Schofield equation	Factorial method		
Stables et al. [31]	In-season	Food photographic diary, 24 h recall	Hannon equation	Doubly labelled water		×
Stables et al. [46]	In-season	Food photographic diary, 24 h recall		Daily record		

NR (not reported).

According to the type of analysis, only two studies considered the effect of playing position on energetic and dietary parameters [35, 49]. The impact of match day or training intensity on energetic and dietary variables was also tested solely into five studies [31, 32–34, 39].

Table 3 summarizes the descriptive statistics (mean ± standard deviation) for energy and macronutrient intake, total energy expenditure, and resting metabolic rate.

**TABLE 3:** Mean and standard deviation of dietary intake and energy expenditure.

Study	Energy intake	Macronutrient intake	Total energy expenditure	Resting metabolic rate
Sanz et al. [30]	3952 ± 1071 kcal · day^−1^ 62 ± 12 kcal · kg^−1^ · day^−1^	CHO: 526 ± 62 g · day^−1^ PRT: 142 ± 17 g · day^−1^ FAT: 142 ± 23 g · day^−1^	3883 ± 571 kcal · day^−1^	

LeBlanc et al. [47]	2754 ± 475 kcal · day^−1^	CHO: 356 ± 67 g · day^−1^ PRT: 1.9 ± 0.4 g · kg^−1^ · day^−1^ FAT: 102 ± 23 g · day^−1^		

Iglesias-Gutiérrez et al. [41]	3003 kcal · day^−1^ 47 kcal · kg^−1^ · day^−1^	CHO: 5.6 ± 67 g · kg^−1^ · day^−1^ PRT: 1.9 g · kg^−1^ · day^−1^ FAT: 127 g · day^−1^	2983 kcal · day^−1^	1563 kcal · day^−1^

Murph and Jeanes [42]	2450 ± 429 kcal · day^−1^	CHO: 310 ± 22 g · day^−1^ PRT: 104 ± 16 g · day^−1^ FAT: 88 ± 4 g · day^−1^		

Caccialanza et al. [43]	2600 ± 625 kcal · day^−1^ 38 ± 10 kcal · kg^−1^ · day^−1^	CHO: 5.0 ± 1.4 g · kg^−1^ · day^−1^ PRT: 1.5 ± 0.4 g · kg^−1^ · day^−1^		

Iglesias-Gutiérrez et al. [44]	2913 kcal · day^−1^ 47 kcal · kg^−1^ · day^−1^	CHO: 6.9 g · kg^−1^ · day^−1^ PRT: 1.9 g · kg^−1^ · day^−1^ FAT: 123 g · day^−1^		

Holway et al. [48]	14 yrs: 3115 ± 835 kcal · day^−1^ 15 yrs: 3368 ± 1219 kcal · day^−1^ 16 yrs: 3662 ± 1106 kcal · day^−1^ 17 yrs: 3371 ± 734 kcal · day^−1^ 18 yrs: 3265 ± 881 kcal · day^−1^ 19 yrs: 3920 ± 1080 kcal · day^−1^	CHO: 6.9 ± 3.3 g · kg^−1^ · day^−1^ PRT: 1.8 ± 0.6 g · kg^−1^ · day^−1^ FAT: 1.6 ± 0.6 g · kg^−1^ · day^−1^ CHO: 6.6 ± 3.2 g · kg^−1^ · day^−1^ PRT: 2.0 ± 0.8 g · kg^−1^ · day^−1^ FAT: 1.8 ± 0.8 g · kg^−1^ · day^−1^ CHO: 7.2 ± 3.5 g · kg^−1^ · day^−1^ PRT: 2.2 ± 0.7 g · kg^−1^ · day^−1^ FAT: 2.1 ± 0.9 g · kg^−1^ · day^−1^ CHO: 6.5 ± 1.7 g · kg^−1^ · day^−1^ PRT: 2.2 ± 0.6 g · kg^−1^ · day^−1^ FAT: 2.4 ± 0.7 g · kg^−1^ · day^−1^ CHO: 5.7 ± 1.9 g · kg^−1^ · day^−1^ PRT: 1.8 ± 0.6 g · kg^−1^ · day^−1^ FAT: 1.7 ± 0.8 g · kg^−1^ · day^−1^ CHO: 6.4 ± 2.2 g · kg^−1^ · day^−1^ PRT: 2.4 ± 0.8 g · kg^−1^ · day^−1^ FAT: 2.3 ± 1.0 g · kg^−1^ · day^−1^	14 yrs: 2910 ± 252 kcal · day^−1^ 14 yrs: 1728 ± 148 kcal · day^−1^ 15 yrs: 3001 ± 272 kcal · day^−1^ 15 yrs: 1766 ± 160 kcal · day^−1^ 16 yrs: 2981 ± 214 kcal · day^−1^ 16 yrs: 1753 ± 126 kcal · day^−1^ 17 yrs: 2986 ± 251 kcal · day^−1^ 17 yrs: 1757 ± 148 kcal · day^−1^ 18 yrs: 3152 ± 249 kcal · day^−1^ 18 yrs: 1854 ± 146 kcal · day^−1^ 19 yrs: 3039 ± 177 kcal · day^−1^ 19 yrs: 1788 ± 104 kcal · day^−1^	

Russel and Pennock [32]	2813 ± 164 kcal · day^−1^ 42 ± 3 kcal · kg^−1^ · day^−1^	CHO: 5.9 ± 0.4 g · kg^−1^ · day^−1^ PRT: 1.7 ± 0.1 g · kg^−1^ · day^−1^ FAT: 1.5 ± 0.1 g · kg^−1^ · day^−1^	3618 ± 61 kcal · day^−1^	1760 ± 28 kcal · day^−1^

Iglesias-Gutiérrez et al. [49]	2794 ± 526 kcal · day^−1^ 39 ± 9 kcal · kg^−1^ · day^−1^	CHO: 4.7 ± 1.1 g · kg^−1^ · day^−1^ PRT: 1.6 ± 0.4 g · kg^−1^ · day^−1^ FAT: 116 ± 30 g · day^−1^		

Briggs et al. [33]	2243 ± 321 kcal · day^−1^	CHO: 5.6 ± 0.4 g · kg^−1^ · day^−1^ PRT: 1.5 ± 0.2 g · kg^−1^ · day^−1^ FAT: 1.2 ± 0.1 g · day^−1^	2550 ± 245 kcal · day^−1^	

Elizondo et al. [50]	2931 ± 117 kcal · day^−1^ 45.7 ± 3.8 kcal · kg^−1^ · day^−1^	CHO: 5.9 ± 0.3 g · kg^−1^ · day^−1^ PRT: 2.1 ± 0.1 g · kg^−1^ · day^−1^ FAT: 1.6 ± 0.1 g · day^−1^	3201 ± 46 kcal · day^−1^	1776 ± 25 kcal · day^−1^

Bettonviel et al. [40]	2938 ± 465 kcal · day^−1^	CHO:6.0 ± 1.5 g · kg^−1^ · day^−1^ PRT: 1.7 ± 0.4 g · kg^−1^ · day^−1^ FAT: 1.2 ± 0.2 g · day^−1^		

Naughton et al. [9]	U13-U14: 1903 ± 432 kcal · day^−1^ U15-U16: 1926 ± 317 kcal · day^−1^ U18: 1958 ± 390 kcal · day^−1^	CHO: 6.0 ± 1.2 g · kg^−1^ · day^−1^ PRT: 2.2 ± 0.5 g · kg^−1^ · day^−1^ FAT: 1.3 ± 0.5 g · kg^−1^ · day^−1^ CHO: 4.7 ± 1.4 g · kg^−1^ · day^−1^ PRT: 1.6 ± 0.3 g · kg^−1^ · day^−1^ FAT: 0.9 ± 0.3 g · kg^−1^ · day^−1^ CHO: 3.2 ± 1.3 g · kg^−1^ · day^−1^ PRT: 2.0 ± 0.3 g · kg^−1^ · day^−1^ FAT: 0.9 ± 0.3 g · kg^−1^ · day^−1^		

Granja et al. [34]	2657 kcal · day^−1^ 37.8 kcal · kg^−1^ · day^−1^	CHO: 5.2 g · kg^−1^ · day^−1^ PRT: 2.1 g · kg^−1^ · day^−1^ FAT: 72 g · day^−1^		

Raizel et al. [35]	41 + 13 kcal · kg^−1^ · day^−1^	CHO: 5.4 ± 1.9 g · kg^−1^ · day^−1^ PRT: 1.9 ± 0.8 g · kg^−1^ · day^−1^ FAT: 1.2 ± 0.3 g · day^−1^		

Ersoy et al. [36]	3225 ± 692 kcal · day^−1^	CHO: 6.3 ± 1.7 g · kg^−1^ · day^−1^ PRT: 1.9 ± 0.5 g · kg^−1^ · day^−1^ FAT: 129 ± 33 g · day^−1^	3322 ± 240 kcal · day^−1^	

Noronha et al. [51]		CHO: 3.9 ± 1.0 g · kg^−1^ · day^−1^ PRT: 1.4 ± 0.5 g · kg^−1^ · day^−1^		

Hannon et al. [37]	U12-U13: 2659 ± 187 kcal · day^−1^ U15: 2821 ± 338 kcal · day^−1^ U18: 3180 ± 279 kcal · day^−1^	CHO: 7.3 ± 1.0 g · kg^−1^ · day^−1^ PRT: 1.9 ± 0.5 g · kg^−1^ · day^−1^ FAT: 2.6 ± 0.4 g · kg^−1^ · day^−1^ CHO: 5.8 ± 0.8 g · kg^−1^ · day^−1^ PRT: 2.1 ± 0.3 g · kg^−1^ · day^−1^ FAT: 2.1 ± 0.4 g · kg^−1^ · day^−1^ CHO: 4.8 ± 0.6 g · kg^−1^ · day^−1^ PRT: 2.1 ± 0.5 g · kg^−1^ · day^−1^ FAT: 1.8 ± 0.4 g · kg^−1^ · day^−1^	2859 ± 265 kcal · day^−1^ 3029 ± 262 kcal · day^−1^ 3586 ± 487 kcal · day^−1^	1892 ± 211 kcal · day^−1^ 2023 ± 165 kcal · day^−1^ 2236 ± 93 kcal · day^−1^

Carter et al. [39]	2598 ± 882 kcal · day^−1^	CHO: 3.7 ± 1.5 g · kg^−1^ · day^−1^		2060 ± 216 kcal · day^−1^

Martinho et al. [38]	1926 ± 388 kcal · day^−1^ 32 ± 7 kcal · kg^−1^ · day^−1^	CHO: 7.3 ± 1.0 g · kg^−1^ · day^−1^ PRT: 2.5 ± 0.4 g · kg^−1^ · day^−1^ FAT: 2.6 ± 0.4 g · kg^−1^ · day^−1^	3568 ± 251 kcal · day^−1^	

Stables et al. [31]	2178 ± 319 kcal · day^−1^ 44 ± 12 kcal · kg^−1^ · day^−1^	CHO: 5.6 g · kg^−1^ · day^−1^ PRT: 1.7 ± 0.6 g · kg^−1^ · day^−1^ FAT: 1.6 ± 0.6 g · kg^−1^ · day^−1^	3380 ± 517 kcal · day^−1^

CHO (carbohydrate); PRT (protein); FAT (fat); U (under); yrs (years).

**FIG. 4:**
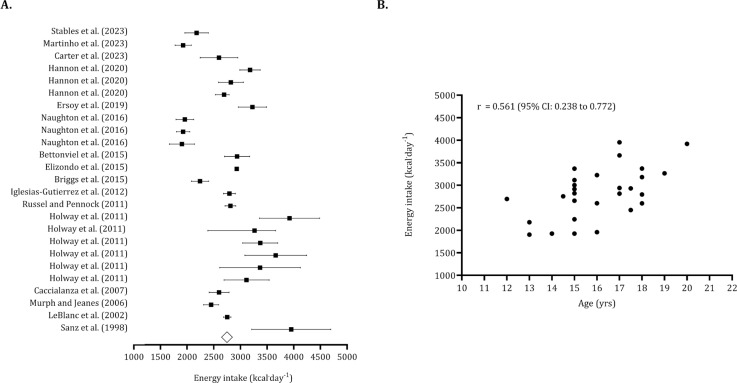
Forest plot for energy intake (panel A) and association between age and energy intake (panel B). Panel A – Black squares: individual studies. White diamond: overall mean. Panel B – Black dots: individual studies. 95% CI (95% confidence intervals).

The overall energy intake, based on means and standard deviations from the pooled sample (*n* = 714), was calculated to be 2,773 kcal · day^−1^ (95% CI: 2,612 to 2,993 kcal · day^−1^), as illustrated in Figure 4 (panel A). Following the identification of five trimmed studies, the estimated overall intake was adjusted to 2,623 kcal · day^−1^ (95% CI: 2,468 to 2,780 kcal · day^−1^). The Egger’s regression intercept was non-significant, indicating no publication bias (Egger’s intercept: -2.005, *p* = 0.203). The association between energy intake and age was large (*r* = 0.561; 95% CI: 0.238 to 0.772) (Figure 4, panel B).

**FIG. 5:**
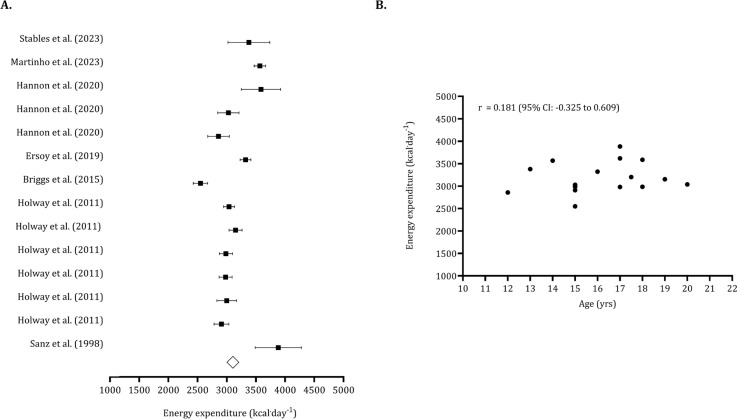
Forest plot for total energy expenditure (panel A) and association between age and energy expenditure (panel B). Panel A – Black squares: individual studies. White diamond: overall mean. Panel B – Black dots: individual studies. 95% CI (95% confidence intervals).

For the data points reporting energy expenditure (*n* pooled = 197), the estimated overall value was 3,134 kcal · day^−1^ (95% CI: 2,978 to 3,291 kcal · day^−1^). No trimmed studies were identified, and the Egger’s intercept was not significant (Egger’s intercept: 0.374, *p* = 0.909), indicating no publication bias (Figure 5, panel A). The association between age and energy expenditure was non-significant (*r* = 0.181, 95% CI: -0.325 to 0.609), as shown in Figure 5 (panel B).

**FIG. 6:**
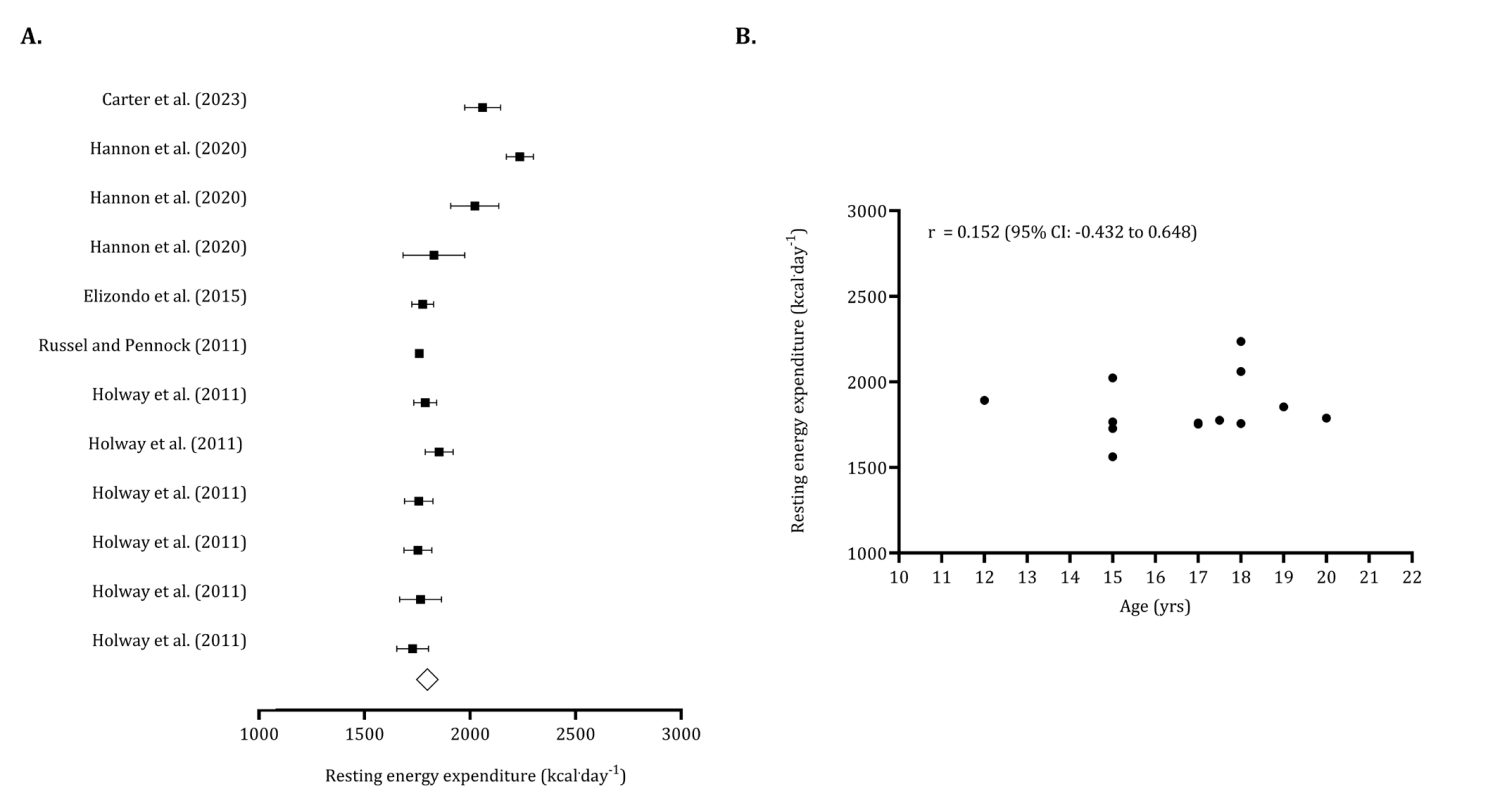
Forest plot for resting energy expenditure (panel A) and association between age and resting energy expenditure (panel B). Panel A – Black squares: individual studies. White diamond: overall mean. Panel B – Black dots: individual studies. 95% CI (95% confidence intervals).

The overall resting energy expenditure, calculated from 222 pooled youth players, was 1,859 kcal · day^−1^ (95% CI: 1,778 to 1,941 kcal · day^−1^). After adjusting for one trimmed study, the overall resting energy expenditure was revised to 1871 kcal · day^−1^. The non-significance of Egger’s regression intercept suggested no publication bias (Egger’s intercept: 3.51, *p* = 0.140). The relationship between age and resting energy expenditure was not significant (*r* = 0.152, 95% CI: -0.432 to 0.648) (Figure 6).

**FIG. 7:**
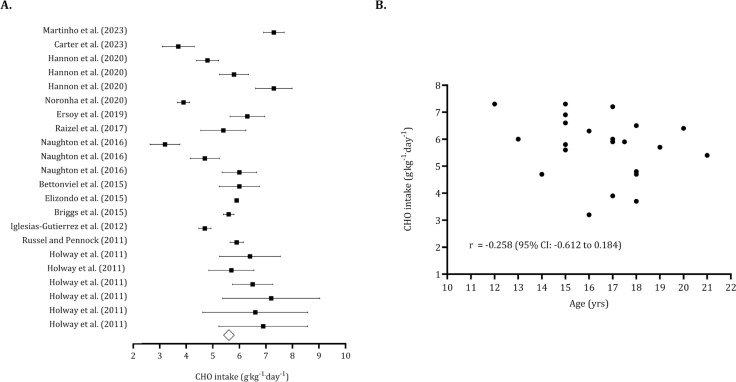
Forest plot for resting carbohydrate intake (panel A) and association between age and carbohydrate intake (panel B). Panel A – Black squares: individual studies. White diamond: overall mean. Panel B – Black dots: individual studies. 95% CI (95% confidence intervals).

**FIG. 8:**
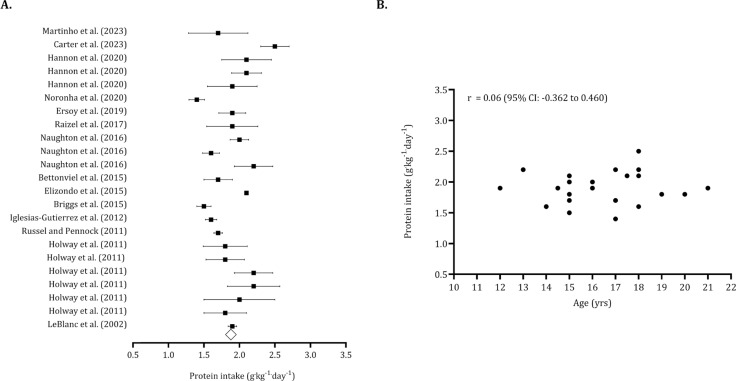
Forest plot for protein intake (panel A) and association between age and protein intake (panel B). Panel A – Black squares: individual studies. White diamond: overall mean. Panel B – Black dots: individual studies. 95% CI (95% confidence intervals).

The overall carbohydrate intake (pooled *n* = 540) was 5.66 g · kg^−1^ · day^−1^ (95% CI: 5.60 to 5.71 g · kg^−1^ · day^−1^). After identifying five trimmed studies, the point estimate for overall carbohydrate intake was adjusted to 5.29 g · kg^−1^ · day^−1^ (95% CI: 4.88 to 5.70 g · kg^−1^ · day^−1^). The Egger’s intercept indicated no publication bias (Egger’s intercept: -1.08, *p* = 0.485). (Figure 7, panel A). Figure 7 (panel B) illustrates that carbohydrate intake tends to decrease with age (*r* = -0.258, 95% CI: -0.612 to 0.184). The overall protein intake (pooled *n* = 694) was 1.88 g · kg^−1^ · day^−1^ (95% CI: 1.76 to 2.00). Six trimmed studies were identified, and the overall value of protein intake was 2.02 g · kg^−1^ · day^−1^ (95% CI: 1.89 to 2.14 g · kg^−1^ · day^−1^). No publication bias was found (Egger intercept: -2.28, *p* = 0.09) (Figure 8, panel A). Protein intake was not associated with age (*r* = 0.06, 95% CI: -0.362 to 0.460). (Figure 8, panel B).

The energy balance was initially found to be negative when considering the values reported in Table 4. After identifying trimmed studies, the energy balance varied between -150 kcal · day^−1^ and -668 kcal · day^−1^. However, when the energy intake was adjusted upward by 15%, the difference between energy intake and energy expenditure became less significant. In this case, the energy balance ranged from -215 kcal · day^−1^ to +327 kcal · day^−1^.

**TABLE 4:** Sensibility analysis using different correlation coefficients to interpret the difference between energy intake and energy expenditure in absolute values and adjusted by 15%.

**Meta-analysis with reported values of energy intake**	**Correlation coefficient, study [24]**	**Energy balance (intake – expenditure)**				**Egger’s regression intercept**		**Duval and Tweedie’s trim and fill**			

		Difference in means	Lower limit	Upper Limit	*p*-value	Intercept	*p*-value	Trimmed studies	Point estimate	Lower limit	Upper Limit

	*r*= 0.893 (Ebbine et al. 2002)	-230	-455	-7	0.043	-0.887	0.794	2	-358	-567	-150

	r = 0.02 (Fudge et al., 2006)	-253	-525	-21	0.070	-0.462	0.825	3	-422	-668	-175

**Meta-analysis with adjusted values of energy intake by 15% (Posluna et al. [23])**	*r*= 0.893 (Ebbine et al. 2002)	289	162	417	0.001	1.957	0.244	2	215	87	343

	r = 0.02 (Fudge et al., 2006)	196	-88	481	0.177	-0481	0.82T6	2	56	-215	327

The descriptive statistics (mean ± standard deviation) for studies that presented energy intake and expenditure, as well as macronutrient composition, are detailed in Supplementary Material 1 and Supplementary Material 2, respectively.

**FIG. 9:**
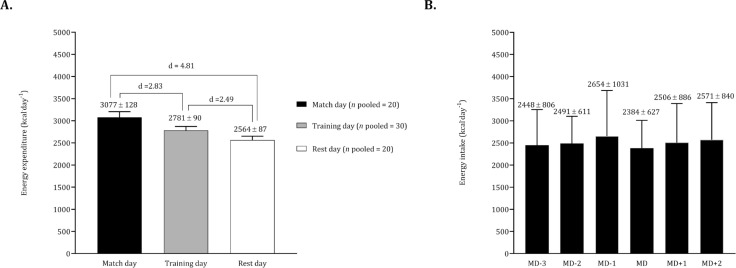
Mean and standard deviation for total energy expenditure according to match, training and rest days (panel A). Mean and standard deviation for energy expenditure according to match day (panel B). For panel A the data of Russel and Pennock [32] and Briggs et al. [33] was pooled (*n* pooled = 20). For panel B the data on match day was calculated by pooling the studies Briggs et al. [33], Carter et al. [39], Stables et al. [31] (*n* pooled = 42) whilst, for the days before and after the match the data of Carter et al. [39] and Stables et al. [31] was combined (*n* pooled = 32).

Considering the studies conducted by Russel and Pennock [32] and Briggs et al. [33], we pooled data on energy expenditure contrasting match days, training days, and rest days. The energy intake of Briggs et al. [33] on match day was pooled with the respective data of Carter et al. [39] and Stables et al. [31]. In light of the training day corresponding to a competitive match, we pooled data from Carter et al. [39] and Stables et al. [31]. The Figure 9 (panel A) illustrates significant differences in energy expenditure, revealing higher values on match days compared to training days. Additionally, energy expenditure on training days was higher than on rest days. In terms of energy intake, elevated values were observed the day before the match, while lower values were recorded on match day (Figure 9, panel B).

Regarding macronutrient intake in relation to the competitive schedule, studies employed various classifications to define training sessions or to report macronutrient consumption. Generally, carbohydrate intake on match day was found to be lower than 6 g · kg^−1^.

### Ergogenic aids

**FIG. 10:**
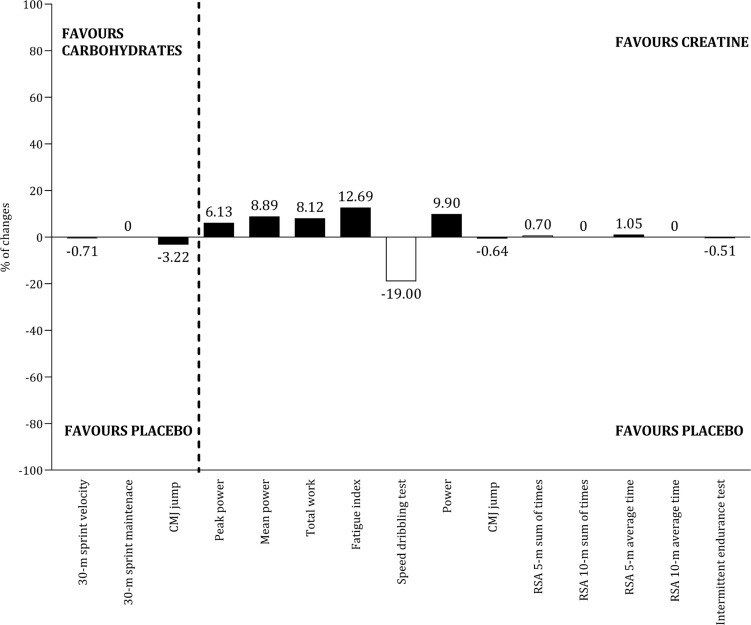
Panel A illustrates the percentage of change in physical capacities between caffeine and placebo, while Panel B compares the percentage of change in physical capacities between carbohydrates and placebo, as well as creatine and placebo.

**FIG. 11:**
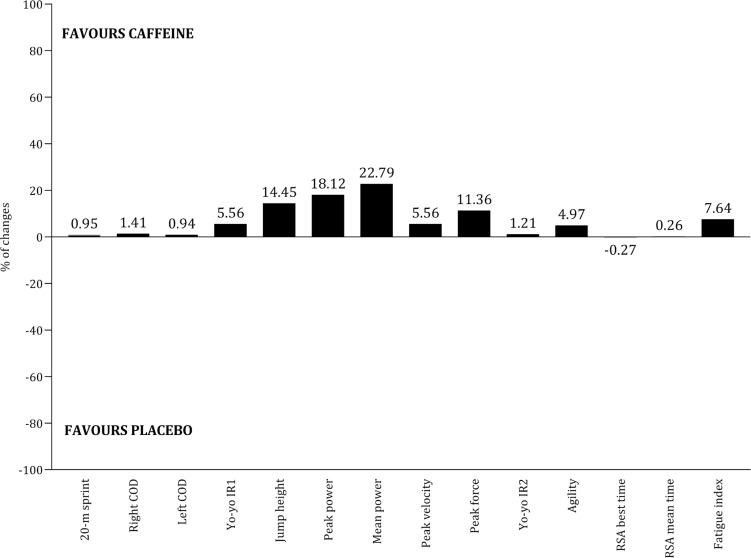
Percentage of chance in technical capacities between caffeine and placebo as well as carbohydrates and placebo.

The effects of seven ergogenic aids on performance were examined: sodium citrate [66], caffeine [56, 58, 59, 62, 65, 69], betaine [64], vitamin D [71], carbohydrates [52, 60, 63, 68], creatine [53, 54, 61, 69], and Resurgex Plus® [55]. Table 5 summarizes the characteristics of the studies involving these ergogenic aids, including dosage, timing, protocol, and main results. Due to variability in performance assessments and the extracted outcomes, conducting a meta-analysis by type of ergogenic aid was not feasible. Meanwhile, Figures 10 and 11 illustrate the effects of the most commonly examined ergogenic aids. In terms of physical capacities, positive effects of caffeine (Figure 10, panel A) and creatine (Figure 10, panel B) were observed across twelve and five outcomes, respectively. However, literature addressing the impact of carbohydrates on physical capacities is limited. Regarding technical and tactical outcomes, the benefits of caffeine and carbohydrates remain inconclusive (Figure 11). The impact of ergogenic aids on physical outcomes (e.g., total distance covered, high-speed running distance) and physiological measures (e.g., heart rate, blood lactate) is limited to a few studies.

**TABLE 5:** Characteristics of studies including ergogenic aids and the main effects on performance outcomes.

Study	Ergogenic aid	Dosage	Timing	Performance protocol	Main findings^*^
Kuru et al. [66]	Sodium Citrate	0.5 g · kg^−1^	3 hours before	Running-based anaerobic test	↑ minimum power output ↑ percentage of decrement score ↔ total sprint time ↔ peak power output ↔ mean power output ↑ rate of perceived exertion

Jafari et al. [65]	Caffeine	3 mg · day^−1^	NR	Video decision task, 10 m short passes, 30 m long pass, Loughborough Soccer Passing Test	↑ short passes ↑ long passes ↔ short passes, decision making, Loughborough Soccer Passing Test score

Nobari et al. [64]	Betaine	2 g · day^−1^	1 hour before and 1 hour after training, 14 weeks	Countermovement jump, 30-m sprint, maximal repetition bench press, maximal repetition squat, running-based anaerobic sprint test, 30–15 intermittent fitness test, COD test	↑ countermovement jump height ↑ 30-m sprint velocity ↑ ${\dot{\text{V}}\text{O}}_{2\max}$ ↑ peak power ↑ strength ↔ COD velocity

Skalska et al. [71]	Vitamin D	5000 IU · day^−1^	8 weeks	Small sided games	↔ physical parameters ↔ heart rate

Ellis et al. [62]	Caffeine	1 mg · kg^−1^, 2 mg · kg^−1^, 3 mg · kg^−1^	1 hour before	20-m sprint, COD test, countermovement jump, Yo-yo IR1	↑ 20-m sprint time ↑ change of direction time ↑ countermovement jump height ↑ Yo-yo distance

Rodríguez- -Giustiniani et al. [63]	CHO	60 g	15 minutes before, half-time	Soccer match stimulation	↔ dribbling accuracy, dribbling speed ↔ sprint ↑ passing accuracy, passing speed ↑ high intensity running

Azevedo et al. [69]	Creatine	0.3 g · kg^−1^ · day^−1^	7 days	High intensity training sessions	↔ heart rate ↔ rating of perceived exertion ↔ blood lactate

Yánez-Silva et al. [61]	Creatine	0.03 g · kg^−1^ · day^−1^	14 days	30-s Wingate test	↑ peak power ↑ mean power ↑ fatigue index ↑ total work

Jastrzębska et al. [70]	Vitamin D	5000 IU · day^−1^	8 weeks	30-s Wingate test, 5-m sprint, 10-m sprint, 20-m sprint, 30-m sprint, squat jump, countermovement jump	↔ peak power ↔ total work capacity ↔ 5-m sprint time ↔ 10-m sprint time ↔ 20-m sprint time ↔ 30-m sprint time ↔ squat jump height ↔ countermovement jump height

Harper et al. [68]	CHO	0.7 g · kg^−1^	5 minutes before extra-time	Soccer match stimulation	↑ dribbling precision ↔ sprint velocity ↔ sprint maintenance ↔ dribbling speed

Russel et al. [60]	CHO	14 ml · kg^−1^ · h^−1^	10 minutes before each half, during the match	Football match	↔ mean heart rate ↔ peak heart rate ↔ blood lactate

Petterson et al. [59]	Caffeine	6 mg · kg^−1^	65 minutes before	Football match, Yoyo IR2	↔ total distance covered ↔ high intensity running ↔ sprinting distance ↔ acceleration counts ↔ heart rate peak percentage ↔ Yo-yo distance

Jordan et al. [58]	Caffeine	6 mg · kg^−1^	1 hour before	Reactive agility test	↑ reaction time nondominant side ↑ rate of perceived exertion ↑ reactive agility test ↔ sprint time, total time

Pereira et al. [56]	Caffeine	6 mg · kg^−1^	1 hour before	Repeated sprint ability	↔ repeated sprint ability best time ↔ mean time ↔ fatigue index

Arent et al. [55]	Resurgex Plus®	NR	20 days, after morning, evening training	Progressive treadmill test	↔ ${\dot{\text{V}}\text{O}}_{2\max}$ ↔ time to exhaustion

Ostojic et al. [54]	Creatine	3 × 10 g doses	7 days	Dribble test, sprint-power test, vertical jump, Luc Léger test	↑ specific dribbling test time ↑ power test time ↑ vertical jump height ↔ shuttle run

Mujica et al. [53]	Creatine	5 g doses · day^−1^	6 days	Countermovement jump, repeated sprint test, intermittent endurance test	↑ repeated sprint time ↔ countermovement jump ↔ intermittent endurance test

Zeederberg et al. [52]	CHO	5 ml · kg^−1^	15 minutes before, half-time	Football match	↔ passing ↔ ball control ↔ success tackling ↔ heading ↔ dribbling ↔ shooting ↔ heart rate

↑: improvements noted by the effect of ergogenic aid; ↔: no differences between groups. Note: the arrows are represented considering the significance level. CHO (carbohydrate); NR (not reported); COD (change of direction); ${\dot{\text{V}}\text{O}}_{2\max}$ (maximal oxygen uptake); Yo-yo IR1 (Yo-yo Intermittent Recovery level 1); Yo-yo IR2 (Yo-yo Intermittent Recovery level 2).

### Risk of bias

Supplementary Table 3 presents the assessment of risk of bias for both observational and interventional studies. In 15 observational studies, the inclusion or eligibility criteria were not clearly described, and only one study provided a justification for its sample size. Dietary and energy outputs were assessed at a single time point in 19 studies. Additionally, five studies did not clearly describe the assessment of energy/nutritional intake or energy expenditure. Using the PEDro scale, interventional studies scored between 5 and 10 points: one study was classified as fair, six studies as good, and the remaining 13 studies as excellent. Allocation concealment was unclear in nine studies, while baseline differences were not described in 13 studies. Furthermore, five studies did not blind the assessors responsible for measuring the outcomes.

## DISCUSSION

The findings from the present review include: 1) the current data provides valuable reference values for nutritionists working with youth soccer players despite variability and potential inaccuracies in methods for estimating dietary intake and energy expenditure, 2) youth soccer players generally exhibit a negative energy balance. However, energy intake may be underestimated by approximately 15%. Adjusting for this underestimation suggests a positive energy balance, indicating that youth soccer players achieve an adequate caloric intake relative to their energy expenditure, 3) carbohydrate intake tends to decrease with age, whereas mean protein intake remains consistent at 1.88 g · kg^−^¹ · day^−^¹ throughout adolescence, 4) energy expenditure is significantly higher on match days compared to training and rest days, 5) on the day preceding a match, energy intake is elevated; however, there is no available data regarding macronutrient intake according to match status, 6) both caffeine and creatine appear to have positive impacts on various physical capacities. In contrast, the effects of carbohydrates on technical performance requires further investigation.

### Energy expenditure and energy intake

The average energy expenditure of youth soccer players identified in this review (3,134 kcal · day^−1^) falls within the range recorded for elite soccer players in the English Premier League, which ranges from 3,047 to 4,400 kcal · day^−1^ [72]. Given that the primary objective of soccer academies is to develop youth players to adult professional teams or to capitalize on their sale, it is unsurprising that youth players are often placed in highly competitive environments [73]. However, it is important to note that the training load parameters do not increase linearly as players progress through competitive age groups. For instance, the cumulative distance covered over a 14-day period was similar for under-15 (53.7 ± 4.5 km) and under-18 (54.4 ± 7.1 km) academy players. Similarly, the speed during this period did not differ significantly between under-12/13 players (67 ± 2 m · min^−1^) and under-15 players (63 ± 4 m · min^−1^) [37]. However, the internal load, which reflects the stress imposed on players, measured as the time spent above 85% and 90% heart rate, was significantly higher in under-15 players compared to older outfield youth soccer players [7]. Given these findings, the assumption that energy expenditure of youth players increases linearly with age should be approached with caution.

The data from the present review confirm that resting energy expenditure tends to stabilize during adolescence [74]. This stabilization impacts total daily energy expenditure, as resting energy expenditure can represent more than 70% of total energy expenditure [75, 76]. Indirect calorimetry assessments also revealed negligible differences in resting energy expenditure between under-12 and under-14 players, as well as between under-15 and under-23 players [77]. The average total daily energy expenditure over 14 days for under-12/13 players (2,859 ± 265 kcal · day^−1^) and under-15 players (3,029 ± 262 kcal · day^−1^) overlaps with the range observed for under-18 players (2,542–5,172 kcal · day^−1^) [37]. Although this review incorporates various methodologies, each with its associated limitations, to estimate overall daily energy expenditure, it offers valuable insights for nutritionists working with youth soccer players, particularly given the uncertainty surrounding the adjustment of energy needs based on age. Notably, longitudinal data are necessary to confirm that energy requirements increase during adolescence, as previous studies, including those aggregated in this review, are primarily based on cross-sectional analyses.

Another limitation of the studies included in this review is the lack of consideration for the biological maturation of youth soccer participants, which may explain the wide range of values observed across different age groups. Consequently, it is crucial to define reference values for resting energy expenditure and total daily energy expenditure within age groups, taking biological maturation of players into account. Gains in fat-free mass during the maximal growth spurt (13–15 years-old) can average approximately 7.2 kg · year^−1^ [78]. This maximal growth spurt in youth soccer players typically occurs between the ages of 11.92 and 15.59, indicating that those who experience the spurt earlier may change fat-free mass sooner than their peers [11]. Fat-free mass is a key predictor of resting energy expenditure and significantly influences daily energy expenditure [77]. Therefore, nutritionists should monitor changes in height, weight, and fat-free mass to better understand the energy requirements of athletes, considering biological maturity rather than relying solely on chronological age. The existing equation for predicting resting energy expenditure in youth soccer players based on fat-free mass requires cross-validation with independent samples. However, the formula to predict resting energy expenditure should be used by nutritionists when indirect calorimetry is not available [77].

The issue of higher energy expenditure relative to energy intake, commonly referred to as under-fuelling, has been reported in youth soccer players [9, 38, 48] and is associated with negative performance and health outcomes. This review found that the overall daily energy intake was substantially lower than daily energy expenditure. However, previous studies have frequently noted the underreporting of dietary intake [24, 43, 79]. When a correction of 15% was applied to daily energy intake, the energy balance appeared positive. Given this context, can the issue of under-fuelling be confounded by the under-reporting linked to self-reported dietary tools? Additionally, studies that assessed daily energy intake indicated a positive association with age, suggesting that, throughout adolescence, youth players tend to increase their energy intake, even as energy expenditure remains relatively stable during this period. The concept of chronically low energy availability in youth athletes, calculated as (exercise energy expenditure – daily energy intake ÷ divided by fatfree mass, defined as < 30 kcal · kg fat-free mass^−1^ · day^−1^, is also associated with negative health outcomes, particularly an increased risk of stress fractures [80]. This concept warrants special consideration in youth athletes due to two main factors: (1) the problem of under-reporting dietary intake, and (2) potential errors in assessing fat-free mass, especially in adolescents, where significant changes occur during the second decade of life. Therefore, relying on a fixed cut-off of 30 kcal · kg fat-free mass^−1^ · day^−1^ to define low energy availability across adolescence warrants further discussion.

### Macronutrient intake and periodized nutrition

Based on a well-designed study assessing energy expenditure in twenty-four male adolescent soccer players from an English Premier League academy, it was suggested that “*relative intakes of carbohydrates (CHO), fat, and protein corresponding to 6–8, 1.5–2.5, and 2 g · kg*^−1^ *· d*^−1^ *of body mass would provide a reasonable starting point to meet the daily energy requirements of academy soccer players*” [37]. However, in this review, the relative intake of carbohydrates was found to be below these recommended guidelines, while the protein and fat intakes met the established recommendations. Nutritionists should investigate whether the under-reporting of energy intake is linked to an underestimation of carbohydrate consumption, or if youth soccer players need to be advised to increase their carbohydrate intake. If the issue arises from under-reporting, nutritionists should adopt an individualized approach that combines various tools, such as food diaries and 24-hour recalls, to enhance the accuracy of nutritional intake assessments [31, 37, 39, 46]. If it is determined that players need to increase their carbohydrate intake, the intervention should be comprehensive, involving parents and managers, as their influence on food preferences and choices of players is significant [15, 81]. Offering practical options to achieve adequate carbohydrate quantities, combined with educational interventions that engage both parents and managers, represents a viable strategy to address the shortfall in carbohydrate intake. This review highlights the importance of developing these strategies, particularly during late adolescence, as carbohydrate consumption tends to decrease with age.

The variation in energy expenditure throughout the week showed that expenditure was significantly higher on match days compared to training and rest days. These findings are consistent with data obtained from GPS/GNSS technologies used in youth soccer academies in England [8] and Italy [7]. In both academies, external load variables such as total distance, velocity, distance covered at > 20 km · h^−1^, the number of accelerations and decelerations were greater on match days than on training days [7, 8]. This suggests that energy and macronutrient intake should be adjusted during the weekly microcycle [72]. However, energy intake was not higher on match days and tended to remain relatively stable throughout the week, indicating that youth soccer players do not effectively periodize their energy intake to match the demands of training and competition. Furthermore, the combination of data for carbohydrate and protein intake was not possible, highlighting a need for future studies in this area. Another important question concerns the impact of nutritional adjustments on performance within the weekly microcycle. In a randomized crossover trial with ten male youth soccer players, training with reduced carbohydrate availability (0 g · kg^−1^) did not significantly affect total distance covered, average speed, high-speed running distance, number of accelerations and decelerations, heart rate, or rate of perceived exertion compared to training with high carbohydrate availability (5.3 g · kg^−1^) [80]. Investigation into periodized nutrition requires improved communication channels and data sharing among all elements of the sport science and nutrition departments [82].

### Ergogenic aids

The physical demands on youth soccer players tend to increase over their developmental progress [7, 37]. The ingestion of ergogenic aids can serve as an essential strategy to enhance performance, even during the adolescent years.

Two studies reported that mid- and late-adolescent players ingested 3 mg · kg^−1^ of caffeine before performance protocols [62, 65], while another two studies used a dosage of 6 mg · kg^−1^ prior to exercise [56, 58], suggesting these quantities may be optimal for improving physical performance. However, the average caffeine intake during 90 minutes of competitive match play in the English Premier League (2.8 ± 1.1 mg · kg^−1^) was below the recommended 3–6 mg · kg^−1^ [83]. This indicates that professional athletes are failing to meet the recommended caffeine intake guidelines. Furthermore, a study included in this review also indicated that a dosage of 3 mg · kg^−1^ positively affected the majority of performance tests, while the benefits of lower dosages (1 and 2 mg · kg^−1^) were not as apparent [62]. During intense training periods, particularly those focused on developing physical capacities in late adolescents, evidence suggests that a caffeine dose of at least 3 mg · kg^−1^ is an effective ergogenic aid.

Although most physical capacities associated with creatine ingestion tend to improve, it is challenging to draw robust conclusions due to variability in interventions regarding dosage and timing. Additionally, further evidence is needed regarding carbohydrate intake, given the lack of studies and the challenges soccer players face in meeting the recommendations of 30–60 g · h^−1^ as proposed by the Union of European Soccer Associations [13, 83].

### Risk of bias

In observational research, it is important to highlight limitations that may impact the conclusions drawn from each study: (1) in fifteen studies, the inclusion criteria did not specify which playing positions were evaluated. Only two studies differentiated participants according to their positional role [35, 49]. The playing position significantly influences the interpretation of physical demands in soccer players [7, 84] and, consequently, should be considered when interpreting energy expenditure and dietary intake. Justification for the sample size was only provided in one study [46], which is particularly problematic for papers comparing energy expenditure and dietary intake within the microcycle. The absence of sample size calculations can lead to statistical bias in the study results [85]. Although twenty studies have examined the use of ergogenic aids in youth soccer players, several methodological issues require revision by researchers. Allocation concealment —where the person determining a subject’s eligibility for inclusion in the trial is unaware of the group allocation— was not reported in approximately 45% of the studies involving ergogenic aids, which can significantly affect the final conclusions. Another issue is the lack of reporting on baseline differences, which is associated with insufficient methodological descriptions of the randomization process [86, 87]. Future intervention studies should specify whether the randomization was simple, block, stratified, or involved covariate adjustments or previous assignment of participants [88]. Additionally, details on sample size calculations should be mandatory, as most studies have utilized small samples, increasing the chance of type II errors —concluding that an ergogenic aid is ineffective when it is actually effective [89].

### Limitations and future research

This review has certain limitations that should be acknowledged. Firstly, the focus was focused solely on youth players at national and international levels, following the approach of Franceschi et al. [7]. Consequently, the findings may not be applicable to recreational players or those from different age groups. During the full-text selection process, some studies lacked clear descriptions of the competitive level. Despite efforts to contact the corresponding authors for clarification, some did not respond, potentially leading to the exclusion of relevant studies. Lastly, a meta-analysis on the effects of ergogenic aids on performance has limitations due to the variability in the methodological procedures across the included studies.

Future studies should investigate the energetic and dietary outcomes in male youth soccer players by following them longitudinally throughout adolescence. Additionally, examining the effects of ergogenic aids within large samples of youth players is essential. To ensure a comprehensive and evidence-based approach, it is important to consider methodological procedures such as allocation concealment, randomization, timing, and dosage. Moreover, the issue of macronutrient periodization and its effects on performance also merits further investigation.

## CONCLUSIONS

Optimising conditions for the development of youth athletes has garnered significant attention within the field of talent development in sport [90, 91]. In youth soccer, nutrition plays a critical role during the adolescent years but has often received less attention than physical, physiological, and technical factors [92].

This review, which examines energy expenditure, dietary intake, and the effects of ergogenic aids, provides valuable insights for nutritionists, soccer clubs, and researchers. The issue of under-fuelling among youth soccer players is particularly concerning, as a positive energy balance was observed after adjusting dietary intake by 15%. Consequently, an individualized approach is essential to assess whether under-reporting of food intake is occurring or if players are meeting nutritional recommendations. In cases of under-reporting, nutritionists should implement educational interventions targeting not only players but also parents and club staff. Additionally, it is crucial for different club departments to share relevant information concerning the energy, physical, and nutritional data of youth players. This collaborative approach ensures a comprehensive understanding of whether players are adhering to nutritional guidelines in line with their weekly microcycle.

Regarding ergogenic aids, both caffeine and creatine have shown positive effects on physical performance, though further research is needed in this area. Future research on optimising athlete nutrition should also focus on leveraging advanced technologies, such as digital platforms, to personalise dietary strategies [93].

Machine learning algorithms could play a key role in analysing physical, physiological, biometric and metabolic data, allowing for precise, real-time adjustments to nutritional requirements. However, it is essential to address the ethical and practical aspects of responsibility when applying artificial intelligence in programming nutritional strategies, ensuring accountability for decision-making processes. Integrating data from physical activity monitoring, dietary intake, and metabolic biomarkers could significantly enhance the implementation of evidence-based nutrition strategies, but further studies are warranted.

In summary, nutritionists should adopt an individualized approach to analyse the energetic and nutritional needs of youth soccer players. Whenever possible, they should also combine different methods to examine dietary intake. Caffeine appears to be beneficial for the performance of youth players, while the acute effects of carbohydrates and creatine in adolescent soccer players require further study.

## Supplementary Material

Nutrition as a missing piece in the development of youth male soccer players: a scoping review and future directions
