# Supplementary material for: Nutrition as a missing piece in the development of youth male soccer players: a scoping review and future directions
Source: Biol Sport. 2025 Sep 9;43:291–317. doi: 10.5114/biolsport.2025.151654 (PMC12884894; doi:10.5114/biolsport.2025.151654)
Supplement: Nutrition as a missing piece in the development of youth male soccer players: a scoping review and future directions [file JBS-43-56161-s1.pdf]

SUPPLEMENTARY MATERIAL

SUPPLEMENTARY TABLE 1. Mean and standard deviation of energy intake and expenditure according to competitive context.

Study	Energetic outputs	Competitive context	Mean ± SD
Russel and Pennock [32]	Total energy expenditure (kcal·day^{-1})	Match day	3880 ± 159
		Training day	3401 ± 124
		Rest day	3047 ± 88
Briggs et al. [33]	Total energy expenditure (kcal·day^{-1})	Heavy training	2870 ± 73
		Moderate training	2434 ± 74
		Rest day	2082 ± 86
		Match day	2733 ± 97
	Energy intake (kcal·day^{-1})	Heavy training	2357 ± 122
		Moderate training	2189 ± 110
		Rest day	2240 ± 74
		Match day	2195 ± 122
Granja et al. [34]	Energy intake (kcal·day^{-1})	Training day	2646
		Match day	2667
Carter et al. [39]	Energy intake (kcal·day^{-1})	Match day -3	2597 ± 843
		Match day -2	2679 ± 641
		Match day -1	2743 ± 1143
		Match day	2582 ± 867
		Match day +1	2580 ± 934
		Match day +2	2714 ± 931
		Match day + 3	2295 ± 817
Stables et al. [31]	Energy intake (kcal·day^{-1})	Match day -4	2214 ± 986
		Match day -3	2000 ± 685
		Match day -2	1928 ± 512
		Match day -1	2386 ± 687
		Match day	2028 ± 486
		Match day +1	2285 ± 729
		Match day +2	2143 ± 543

SD (standard deviation).

SUPPLEMENTARY TABLE 2. Mean and standard deviation of macronutrients intake according to competitive schedule.

Study	Macronutrient intake	Competitive schedule	Mean ± SD
Briggs et al. [33]	Carbohydrate ($g \cdot kg^{-1} \cdot day^{-1}$)	Heavy training	6.0 ± 2.3
		Moderate training	5.6 ± 1.6
		Rest day	5.0 ± 1.3
		Match day	5.5 ± 2.0
	Protein ($g \cdot kg^{-1} \cdot day^{-1}$)	Heavy training	1.6 ± 0.5
		Moderate training	1.4 ± 0.6
		Rest day	1.7 ± 0.5
		Match day	1.5 ± 0.5
	Fat ($g \cdot kg^{-1} \cdot day^{-1}$)	Heavy training	1.3 ± 0.4
		Moderate training	1.1 ± 0.5
		Rest day	1.4 ± 0.3
		Match day	1.1 ± 0.2
Granja et al. [34]	Carbohydrate ($g \cdot kg^{-1} \cdot day^{-1}$)	Training day	5.2
		Match day	5.2
	Protein ($g \cdot kg^{-1} \cdot day^{-1}$)	Training day	2.0
		Match day	2.1
	Fat ($g \cdot kg^{-1} \cdot day^{-1}$)	Training day	1.0
		Match day	1.0
Carter et al. [39]	Carbohydrate ($g \cdot kg^{-1} \cdot day^{-1}$)	Match day -3	3.5 ± 1.5
		Match day -2	3.5 ± 1.1
		Match day -1	3.9 ± 1.9
		Match day	4.2 ± 1.6
		Match day +1	3.6 ± 1.7
		Match day +2	4.0 ± 2.0
		Match day + 3	3.3 ± 1.5
Stables et al. [31]	Carbohydrate ($g \cdot day^{-1}$)	Match day -4	297 ± 144
		Match day -3	237 ± 77
		Match day -2	246 ± 57
		Match day -1	323 ± 82
		Match day	294 ± 77
		Match day +1	271 ± 78
		Match day +2	297 ± 86
	Protein ($g \cdot day^{-1}$)	Match day -4	94 ± 38
		Match day -3	81 ± 37
		Match day -2	76 ± 30
		Match day -1	89 ± 30
		Match day	85 ± 45
		Match day +1	87 ± 43
		Match day +2	89 ± 28

SD (standard deviation).

SUPPLEMENTARY MATERIAL 3. Questions from the Quality Assessment Tool for Observational Cohort and Cross-Sectional Studies checklist used to evaluate the methodological quality of the included articles.

Criteria no.	Question
1	Was the research question or objective in this paper clearly stated?
2	Was the study population clearly specified and defined?
3	Was the participation rate of eligible persons at least 50%?
4	Were all the subjects selected or recruited from the same or similar populations (including the same time period)? Were inclusion and exclusion criteria for being in the study prespecified and applied uniformly to all participants?
5	Was a sample size justification, power description, or variance and effect estimates provided?
6	For the analyses in this paper, were the exposure(s) of interest measured prior to the outcome(s) being measured?
7	Was the timeframe sufficient so that one could reasonably expect to see an association between exposure and outcome if it existed?
8	For exposures that can vary in amount or level, did the study examine different levels of the exposure as related to the outcome (e.g., categories of exposure, or exposure measured as continuous variable)?
9	Were the exposure measures (independent variables) clearly defined, valid, reliable, and implemented consistently across all study participants?
10	Was the exposure(s) assessed more than once over time?
11	Were the outcome measures (dependent variables) clearly defined, valid, reliable, and implemented consistently across all study participants?
12	Were the outcome assessors blinded to the exposure status of participants?
13	Was loss to follow-up after baseline 20% or less?
14	Were key potential confounding variables measured and adjusted statistically for their impact on the relationship between exposure(s) and outcome(s)?

Legend:
1 – YES; 2 – NO; 3 – cannot determine, not applicable or not reported

SUPPLEMENTARY MATERIAL 3. Results of methodological quality assessment for Obs1ervational Cohort and Cross-Sectional Studies.

Study	Quality Assessment Tool for Observational Cohort and Cross-Sectional Studies checklist question number													
	1	2	3	4	5	6	7	8	9	10	11	12	13	14
Sanz et al. [30]	1	1	3	2	2	3	3	3	1	2	1	3	3	1
LeBlanc et al. [47]	1	2	3	2	2	3	3	3	1	1	1	3	3	1
Iglesias-Gutiérrez et al. [41]	1	1	3	2	2	3	3	3	1	2	1	3	3	1
Murph and Jeanes [42]	1	1	3	2	2	3	3	3	1	2	2	3	3	2
Caccialanza et al. [43]	1	1	3	2	2	3	3	3	1	1	1	3	3	1
Iglesias-Gutiérrez et al. [44]	1	1	3	2	2	3	3	3	1	2	1	3	3	1
Holway et al. [48]	1	1	3	2	2	3	3	3	1	2	2	3	3	1
Russel and Pennock [32]	1	1	3	1	2	3	3	3	1	2	1	3	3	1
Iglesias-Gutiérrez et al. [49]	1	1	3	2	2	3	3	3	1	2	1	3	3	1
Briggs et al. [33]	1	1	3	1	2	3	3	3	1	2	1	3	3	1
Elizondo et al. [50]	1	1	3	2	2	3	3	3	1	2	1	3	3	1
Naughton et al. [9]	1	1	3	1	2	3	3	3	1	2	1	3	3	1
Granja et al. [34]	1	1	3	2	2	3	3	3	1	2	2	3	3	1
Hosseinzadeh et al. [45]	1	1	3	2	2	3	3	3	1	2	2	3	3	1
Raizel et al. [35]	1	1	3	1	2	3	3	3	1	2	1	3	3	1
Ersoy et al. [36]	1	1	3	2	2	3	3	3	1	2	2	3	3	1
Noronha et al. [51]	1	1	3	1	2	3	3	3	1	2	1	3	3	1
Hannon et al. [37]	1	1	3	1	2	3	3	3	1	2	1	3	3	1
Carter et al. [39]	1	1	3	2	2	3	3	3	1	1	1	3	3	1
Martinho et al. [38]	1	1	3	2	2	3	3	3	1	2	1	3	3	1
Stables et al. [31]	1	1	3	1	1	3	3	3	1	2	1	3	3	2
Stables et al. [46]	1	1	3	2	2	3	3	3	1	2	1	3	3	1

SUPPLEMENTARY MATERIAL 3. Questions from the PEDro scale used to evaluate the methodological quality of the interventional studies.

PEDro scale
1. eligibility criteria were specified
2. subjects were randomly allocated to groups (in a crossover study, subjects were randomly allocated an order in which treatments were received)
3. allocation was concealed
4. the groups were similar at baseline regarding the most important prognostic indicators
5. there was blinding of all subjects?
6. there was blinding of all therapists who administered the therapy
7. there was blinding of all assessors who measured at least one key outcome
8. measures of at least one key outcome were obtained from more than 85% of the subjects initially allocated to groups
9. all subjects for whom outcome measures were available received the treatment or control condition as allocated or, where this was not the case, data for at least one key outcome was analysed by “intention to treat”
10. the results of between-group statistical comparisons are reported for at least one key outcome
11. Were the outcome measures (dependent variables) clearly defined, valid, reliable, and implemented consistently across all study participants?
**Total points**

SUPPLEMENTARY MATERIAL 3. Results of methodological quality assessment for PEDro scale.

Study	Quality Assessment Tool for PEDro scale												Qualitative classification
	1	2	3	4	5	6	7	8	9	10	11	Points	
Zeederberg et al. [52]	0	0	0	0	0	0	0	1	1	1	1	**4**	**Fair**
Mujika et al. [53]	1	1	0	1	1	1	1	1	1	1	1	**9**	**Excellent**
Ostojic et al. [54]	0	1	0	1	0	0	0	1	1	1	1	**6**	**Good**
Arent et al. [55]	1	1	0	0	1	0	0	1	1	1	1	**7**	**Good**
Pereira et al. [56]	0	1	1	0	1	1	1	1	1	1	1	**9**	**Excellent**
Russel et al. [57]	0	1	1	0	1	1	1	1	1	1	1	**9**	**Excellent**
Bortolotti et al. [67]	1	1	1	0	1	1	1	1	1	1	1	**9**	**Excellent**
Jordan et al. [58]	0	1	1	1	1	1	1	1	1	1	1	**9**	**Excellent**
Petterson et al. [59]	1	1	1	0	1	1	1	1	1	1	1	**9**	**Excellent**
Russel et al. [60]	0	1	1	0	1	1	1	1	1	1	1	**9**	**Excellent**
Harper et al. [68]	0	1	1	0	1	1	1	1	1	1	1	**9**	**Excellent**
Jastrzębska et al. [70]	0	1	0	1	1	1	1	1	1	1	1	**9**	**Excellent**
Yãnez-Silva et al. [61]	0	1	0	1	1	1	1	1	1	1	1	**9**	**Excellent**
Azevedo et al. [69]	0	0	0	1	1	0	0	1	1	1	1	**6**	**Good**
Ellis et al. [62]	0	1	1	1	1	0	0	1	1	1	1	**8**	**Good**
Rodriguez-Giustiniani et al. [63]	1	1	1	0	1	1	1	1	1	1	1	**9**	**Excellent**
Skalska et al. [71]	0	0	0	1	1	1	1	1	1	1	1	**8**	**Good**
Nobari et al. [64]	1	1	0	0	1	1	1	1	1	1	1	**6**	**Good**
Jafari et al. [65]	0	1	1	0	1	1	1	1	1	1	1	**9**	**Excellent**
Kuru et al. [66]	1	1	1	0	1	1	1	1	1	1	1	**9**	**Excellent**

Articles published in the Biology of Sport are licensed under an open access Creative Commons CC BY 4.0 license.
